# Carboxymethyl starch as a reducing and capping agent in the hydrothermal synthesis of selenium nanostructures for use with three-dimensional-printed hydrogel carriers

**DOI:** 10.1098/rsos.230829

**Published:** 2023-10-11

**Authors:** Vishakha Vishakha, A. M. Abdel-Mohsen, Jan Michalicka, Paul B. White, Petr Lepcio, Lizeth Katherine Tinoco Navarro, Josef Jančář

**Affiliations:** ^1^ Central European Institute of Technology, Brno University of Technology, Purkyňova 123, Brno, Czech Republic; ^2^ Czech Academy of Sciences, Institute of Macromolecular Chemistry Heyrovského nám. 2, Praha 16206, Czech Republic; ^3^ Institute for Molecules and Materials, Radboud University, PO Box 9010, 6500, GL, Nijmegen, The Netherlands

**Keywords:** carboxymethyl starch, nanostructures, tailored-made polymer, nanomaterials, three-dimensional-printed selenium nanostructures composite

## Abstract

The hydrothermal method is a cost-effective and eco-friendly route for preparing various nanomaterials. It can use a capping agent, such as a polysaccharide, to govern and define the nanoparticle morphology. Elemental selenium nanostructures (spheres and rods) were synthesized and stabilized using a tailor-made carboxymethyl starch (CMS, degree of substitution = 0.3) under hydrothermal conditions. CMS is particularly convenient because it acts simultaneously as the capping and reducing agent, as verified by several analytical techniques, while the reaction relies entirely on green solvents. Furthermore, the effect of sodium selenite concentration, reaction time and temperature on the nanoparticle size, morphology, microstructure and chemical composition was investigated to identify the ideal synthesis conditions. A pilot experiment demonstrated the feasibility of implementing the synthesized nanoparticles into vat photopolymerization three-dimensional-printed hydrogel carriers based on 2-hydroxyethyl methacrylate (HEMA). When submersed into the water, the subsequent particle release was confirmed by dynamic light scattering (DLS), promising great potential for use in bio-three-dimensional printing and other biomedical applications.

## 1. Introduction

Polysaccharides are promising candidates for various fields, including material science, medicine and biotechnology, due to their biodegradability, renewability and versatility. Recently, many studies reported on polysaccharides for drug carrier applications due to their biocompatibility and active hydroxyl groups suitable for chemical modification [1–3]. Starches, among them, are abundant, renewable and inexpensive [4,5]. They consist of amylose and amylopectin chains [6]. Starch is poorly soluble in water at room temperature due to the strong hydrogen bonds of hydroxyl groups, limiting its applications in food, cosmetics, medicine, absorbents and adhesives. However, it could be chemically modified due to the three active hydroxyl positions at C2, C3 and C6 [7,8]. Carboxymethyl starch and its derivatives represent a favoured sub-group in this category. Since 1924, when it was first prepared, it was synthesized from different sources: potato, amaranth, rice and mung bean. These starches vary in their amylose/amylopectin content, causing a varying degree of substitution (DS). Carboxymethyl starch (CMS) could be prepared by starch reaction with sodium monochloroacetate (SMCA) or monochloroacetatic acid (MCA) in the presence of NaOH. Generally, this reaction is performed in a heterogeneous medium (ethanol/water, isopropyl alcohol/water, benzene/water). The reaction between starch and SMCA follows the Williamson ether synthesis and is based on the S_N_2 mechanism. The polar aprotic solvent is required for efficient etherification. DS is controlled by factors such as solvent type, NaOH and SMCA concentration, temperature and reaction time [9,10]. Isopropyl alcohol aqueous solution was previously identified as the most effective solvent for CMS production, giving the best DS. The solubility in water appears already at DS of 0.1 [11–13]. Isopropyl alcohol/water mixture is also convenient for removing unreacted impurities from CMS [14,15].

The active functional groups in polysaccharides such as hydroxyl (-OH), amine (-NH), or carboxylic (-COOH) could template the growth of various nanostructures, such as cages, tubes, rods, springs, etc. Among them, one-dimensional nanomaterials are of extraordinary importance because of their low percolation threshold and high aspect ratio. These could be potentially used in optoelectronics and electronics. One-dimensional nanostructures have been synthesized by known routes such as vapour-liquid-solid growth [16], a hard template limited approach [17] and a surfactant-assisted technique [18].

The strong electron–donor tendency of selenium is markedly improved on the nanoscale [19]. Hence, the elemental form of selenium is highly demanded in chemistry, physics and biology. Its naturally appearing polymorphs are amorphous, trigonal or monoclinic crystalline. The black trigonal selenium is the most stable crystalline form at room temperature. The monoclinic form is red and contains S_8_ rings [20]. Amorphous red, black and vitreous selenium represent non-crystalline forms [21,22]. A recent review paper summarizes different methods and reducing agents to form selenium Se (0) nanostructures with different controllable morphologies [22]. It includes various shapes, such as rods, spheres and cubes, obtained using reducing agents such as bovine serum albumin, D-glucose and soluble starch (amylum), respectively [4,23]. Another approach synthesized selenium nanospheres and nanorods using L-cysteine as a reducing agent [4,24] and polysaccharides as a capping agent. However, only a few studies conducted Se synthesis by hydrothermal method with biopolymers. Among them, selenium nanobelts with a unique ribbon-like structure were synthesized by cellulose templating [25].

Moreover, the time-dependent transformation of α-Se nanospheres to crystalline t-Se nanostructure was identified by microscopy and spectroscopy [26]. Nanostructures are often embedded in a polymer matrix, which may, among others, serve as a carrier for medical applications. Final samples could be shaped by techniques such as vat photopolymerization three-dimensional printing, using light to selectively polymerize a photosensitive resin, which is repeated layer-by-layer to print the final object [27–31].

Our preliminary results proved that carboxymethyl starch could be used to synthesize Se nanoparticles by hydrothermal method [14,15]. CMS is particularly convenient because it acts simultaneously as the capping and reducing agent. This study used CMS with a DS of 0.3 as a reducing and stabilizing agent to investigate the hydrothermal process in more detail. It explored the effect of processing parameters, such as reaction time, temperature and reagent concentration, on the nanoparticle size and morphology. The formation of selenium nanostructures was monitored via colour change (UV-visible (UV-VIS) spectroscopy), dynamic light scattering (DLS), scanning and transmission electron microscopy (SEM and TEM), Fourier transform Infrared spectroscopy (FTIR), X-ray diffraction (XRD) and X-ray photoelectron spectroscopy (XPS). Particular emphasis was put on anisotropic Se nanorods for their potential use in biomedical applications. On top of that, we also included an application case by embedding the Se nanoparticles into a three-dimensional-printed 2-hydroxyethyl methacrylate (HEMA) hydrogel carrier. We monitored their subsequent release into the water, demonstrating their potential to be delivered on the target site and marking the direction of possible further research.

## 2. Experimental section

### 2.1. Materials

Sodium selenite (Na_2_SeO_3_), HEMA, and SMCA were purchased from Sigma Aldrich (Germany) and used as received. Potato starch was obtained from Agrana (Austria). Diphenyl (2,4,6-trimethylbenzoyl)-phosphine oxide (TPO) photoinitiator was obtained from RAHN (Switzerland). CRODA (France) kindly provided the Tween 20 surfactant. Acetone, ethanol, isopropanol and monochloroacetatic acid were purchased from Lach-ner (Czech Republic) and used as received.

### 2.2. Synthesis process

**Scheme 1.**
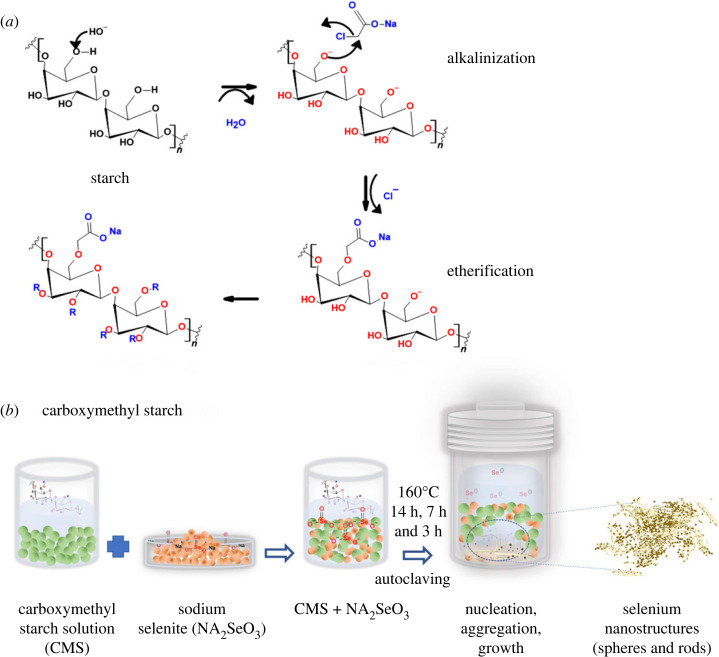
(*a*) Mechanism of the carboxymethyl starch preparation and (*b*) experimental scheme of selenium nanostructures formation.

Carboxymethyl starch was prepared using sodium monochloroacetate, where 1 g (6 mmol) of potato starch was dispersed in 100 ml of the round bottom flask containing 50 ml of isopropyl alcohol-water mixture (9 : 1). The mixture was treated with 1 ml 10% NaOH at room temperature (RT) for 1 h to deprotonate the alcohol groups present in the monomeric units of starch (scheme 1*a*). The mixture was stirred for up to 5 h at RT, followed by adding a sodium monochloroacetate solution (2.096 g/18 mmol in 5 ml of distilled water) and stirring for 2 h at 50°C. After the reaction, a white solid was collected, dried at 50°C for 14 h, and neutralized by 0.4 ml of 6 M HCl for 2 h at RT. Several filtrations with an acetone-water mixture were conducted to purify the product [15,32,33]. The obtained solids were weighed and used to grow selenium nanostructures using the hydrothermal technique. Initially, 0.1 g (1%) of carboxymethyl starch (DS = 0.3, pH 5–6) and 0.1 g (1%) of Na_2_SeO_3_ were dissolved in 10 ml of distilled water [25] and placed into a Teflon-lined stainless steel autoclave. The reaction was performed at 160°C for 14, 7 or 3 h (scheme 1*b*). It was then cooled to room temperature, and the solid content was separated by centrifuging and washed with ethanol. Different Se ion concentrations were obtained by varying the concentration of sodium selenite (1%, 0.5% and 0.25%).

### 2.3. Characterization

#### 2.3.1. X-ray diffraction

X-ray diffraction was measured by Rigaku Smart lab 3 kW (Japan) X-ray powder diffractometer with an automatic ⊖/⊖ goniometer on the solid product purified product placed on a glass slide holder for X-ray measurement. The use of additional series complements the Bragg–Brentano and parallel beam modes. Diffractometer measurement has been taken at 40 kV and a current of 30 mA using a Cu K*α* (*λ* = 0.154 nm).

#### 2.3.2. Scanning electron microscopy

The selenium nanocrystals' morphology, size and organization behaviour were determined by VERIOS 460 L (Thermo Fisher Scientific, USA) and Mira 3 XMU (Tescan, Czech Republic) field-emission scanning electron microscopes. The samples for SEM were prepared by a drip casting of a few microlitres of dispersed nanoparticles in isopropyl alcohol/water on a 200 copper mesh grid with an amorphous carbon holey membrane. To avoid charging, the dried grid was sputter coated with approximately 20 nm Au/Pd layer with ACE600 coater (Leica, Germany). SEM observations were conducted using secondary electron (SE) detectors and in scanning TEM (STEM) mode with dark-field detectors available on the microscopes. The SEM elemental analysis of the selenium nanocomposite was carried out by energy dispersive X-ray spectrometry (EDS) using X-Max 20 spectrometer (Oxford Instruments, UK) attached to the Tescan Mira 3 XMU SEM.

#### 2.3.3. Transmission electron microscopy

TEM analysis of Se nanorod crystal structure and chemical composition were obtained with spherical aberration image corrected transmission electron microscope TITAN Themis 60–300 (Thermo Fisher Scientific, USA) operated at 60 kV and equipped with a Super-X EDS spectrometer. The TEM sample was prepared the same way as for SEM but not coated. TEM data were acquired and processed with SW Velox v. 2.14.

#### 2.3.4. UV-visible spectroscopy

The optical properties of the obtained nanosuspension were investigated by UV-visible spectroscopy with an S-220 spectrophotometer and Jasco V-770 spectrometer in a 10 mm cuvette at spectral range 200–800 nm, and wavelength steps 2 nm.

#### 2.3.5. Dynamic light scattering measurement

Dynamic light scattering was measured with a Zetasizer Ultra instrument from Malvern Panalytical (UK). The hydrodynamic volume and its distribution were calculated using the OEM software. All samples were measured freshly synthesized as obtained without dilution.

#### 2.3.6. Fourier transform infrared measurement

FTIR measurements were performed using an attenuated total reflection Fourier transform infrared (ATR-FTIR) spectrometer (Bruker Vertex 70 V, Germany). The samples were thoroughly dried. The obtained powder was placed on a crystal's surface and held in place with a clutch-type lever before measuring the transmittance. Each sample spectrum was collected from 400 to 4000 cm^−1^ and 128 scans in the wavenumber range.

#### 2.3.7. Thermogravimetry analysis

Thermogravimetry analysis (TGA) measurements were obtained using TGA Discovery (TA instruments, USA). This method shows the changes in the specimen's weight as the temperature increases. Each measurement was taken under the same conditions under a nitrogen atmosphere by ramping the temperature at 10°C min^−1^ up to 700°C, followed by a 5 min isothermal hold.

#### 2.3.8. X-ray photoelectron spectroscopy

XPS measurements of all nanostructures were obtained using an X-ray photoelectron spectrometer Axis Supra (Kratos Analytical, UK). All powdered samples were placed on a double-sided copper tape and inserted into the sample mount. Analysis was carried out with an aluminum monochromator source with one analysis point per sample. Scans were collected between 1200 to 0 eV and 4 mA emission current with a step size from 1 to 0 eV. High-resolution spectra were collected for O1s, C1s, Se 3d with three sweeps, and Na 1 s with two sweeps. The fitting of individual elements was performed using the Casa XPS software (v. 2.3.17) by applying a Gaussian line shape for fitting and the ORIGIN 2016 software.

#### 2.3.9. ^1^H,^13^C nuclear magnetic resonance

All nuclear magnetic resonance (NMR) experiments were performed at 35°C with a JEOL 500 MHz ECZ-R equipped with a RoyalHFX probe. Quantitative ^1^H experiments were run with 32 scans and a total relaxation time (acquisition and relaxation delay) of 23.5 s. The ^1^H data was processed in MestreNova 14 using an exponential line broadening and an ablative baseline correction. Integrals were obtained by performing line fitting across the spectral regions so that H7, which overlaps with the other protons, could be better compared with H1 and H1′. Quantitative ^13^C experiments were performed with the inverse gated decoupling sequence using 5000 scans and a total relaxation time of 50.8 s. ^13^C data were processed using a 30 Hz exponential line broadening and an ablative baseline correction. Integrals were obtained by directly integrating the spectrum.

#### 2.3.10. Three-dimensional-printed hydrogel carriers

The pilot experiment for the three-dimensional printed hydrogel enriched with the selenium nanostructures was carried out with the Original Prusa SL1S 3D printer (Prusa, Czech Republic). It is based on masked stereolithography (M-SLA) technology and equipped with a 405 nm UV LED source (2.07 mW cm^−2^). The resin was prepared by dissolving 0.3 g of TPO in 8 g of HEMA at RT and adding 2 g of Tween 20 and 2 g of purified selenium nanoparticles dispersed in deionized water. The resin was mixed at RT for 10 min, sonicated for 5 min, and filtered using a syringe filter of 0.45 µm. A rectangular beam (2 × 5 × 48.5 mm^3^) was printed with an exposure time of 40/20 s for the first/all other layers and a layer thickness of 25 µm [27]. The printed hydrogel was submerged in water for 21 h, and the nanoparticle extraction was confirmed by dynamic light scattering (Zetasizer Ultra, Malvern Panalytical, UK).

## 3. Results and discussion

### 3.1. Carboxymethyl starch

**Figure 1.**
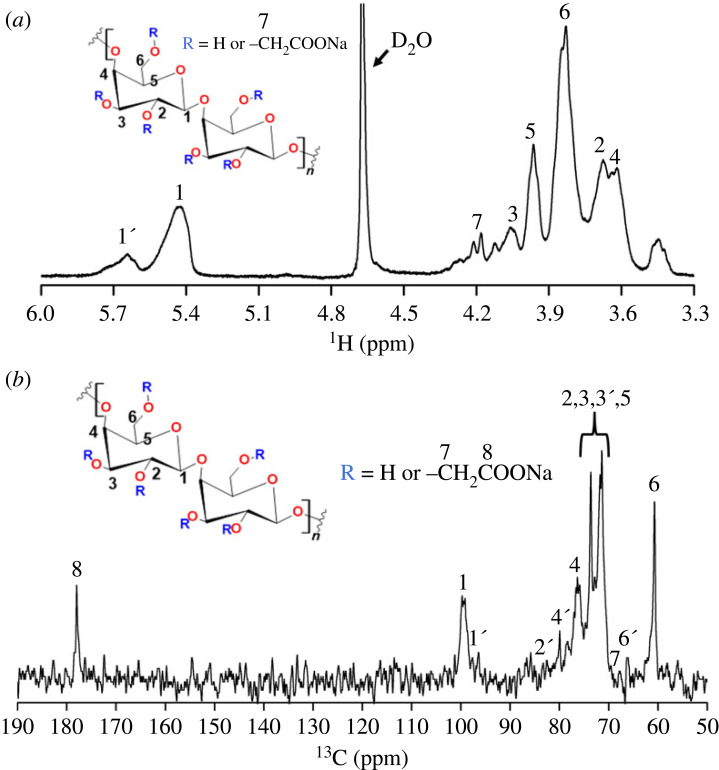
Structural characterization of carboxymethyl starch (*a*) ^1^H NMR spectra of carboxymethyl starch and (*b*) ^13^C NMR spectra of carboxymethyl starch.

The structure of the synthesized carboxymethyl starch was confirmed using ^1^H-NMR (figure 1*a*) and ^13^C NMR (figure 1*b*). The assignments of various carbon types’ resonances, evaluated according to the literature [34,35], are shown directly in the spectra. The position of signal ‘**7**’ corresponding to methylene protons in the carboxymethyl group was found based on the multiplicity edited ^1^H-^13^C heteronuclear single quantum coherence (HSQC) NMR spectrum shown in electronic supplementary material, figure S1. Additionally, H1′ signal appears due to substituting the carboxymethyl group at the O-2 position. ^13^C NMR spectrum yielded line widths typical for a natural amorphous polysaccharide with a broadband signal between 60 and 90 ppm arising from the bulk of the ring, C-OH. C-4 carbon accounts for the high-frequency shoulder, while C-1 between 90 and 110 ppm was attributed to anomeric carbon. The shape of this band suggests that it is composed of multiple signals. ^13^C NMR carboxymethylated starch signal at 178 ppm was assigned to the carbonyl carbon of the carboxymethyl groups (figure 1*b*). The signals marked with a prime (‘) are related to carbons next to substituted hydroxyl groups. The appearance of these signals suggests substitution on all three possible hydroxyl groups. The degree of substitution was calculated using ^13^C NMR spectrum to DS = 0.3 [35].

**Figure 2.**
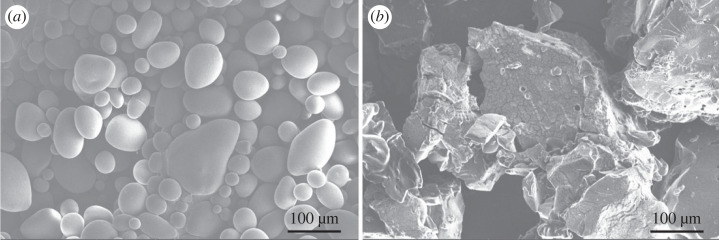
SEM images, morphological characterization of potato starch granules (*a*) before and (*b*) after the chemical modification.

Additionally, the morphology of starch granules changed significantly after the chemical substitution of the carboxymethyl group, shown in figure 2*a,b*. SEM image suggests the approximate granule's size before and after the modification as 10–20 and 200–350 microns, respectively. On the other hand, some granules appear broken after modification. Moreover, the smoothness of the surface was reduced (figure 2*b*). That may reflect the loss of crystallinity after substituting the carboxymethyl group revealed by the XRD results in electronic supplementary material, figure S12.

### 3.2. Reaction time

**Figure 3.**
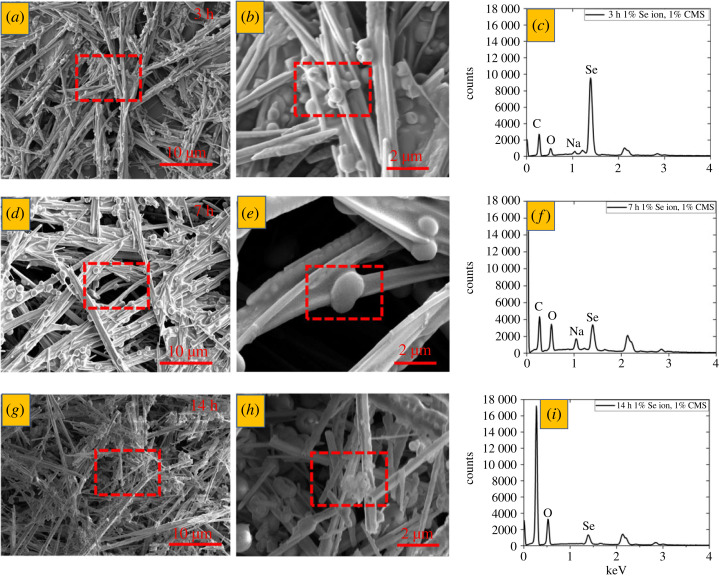
SEM-EDS analysis of selenium nanostructures (spheres, rods) with 1% Na_2_SeO_3_ and 1% CMS. (*a,d,g*) lower and (b,e,h) higher magnification SEM images and (*c,f, i*) EDS spectra for samples after (*a,b,c*) 3 h, (*d,e,f*), 7 h and (*g,h,i*) 14 h of reaction. Red rectangles show the area of larger magnification (*a,d,g*) or EDS spectra collection (*b,e,h*).

In the initial set of experiments, selenium nanostructures (rods, spheres) were prepared hydrothermally using 1% of sodium selenite (Na_2_SeO_3_) as a source of Se ions and 1% of carboxymethyl starch as a reducing agent (figure 3). The reaction was kept at 160°C to activate the functional groups present in the carboxymethyl starch. The mixture was cooled down after 3, 7, or 14 h to cease the reaction. The reaction mixture colour changed from colourless to light orange supernatant and solid precipitate with different shades of black depending on the reaction time. That indicates that the redox reaction occurred in the liquid phase containing monoclinic selenium while the precipitate contained a more stable trigonal polymorph [22,36]. SEM-EDS characterization was performed to analyse the particles and confirm their atomic composition (figure 3). It reveals the effect of time on the growth of selenium nanostructure (rods), which is uniformly distributed with the nanospheres. The presence of carbon, oxygen and sodium next to selenium in the EDS spectra hints at carboxymethyl starch residues in the particles (figure 3*c,f,i*). The Se content decreased as the reaction proceeded. Therefore, the shortest tested time of 3 h yielded the highest Se concentration in the nanostructures.

**Figure 4.**
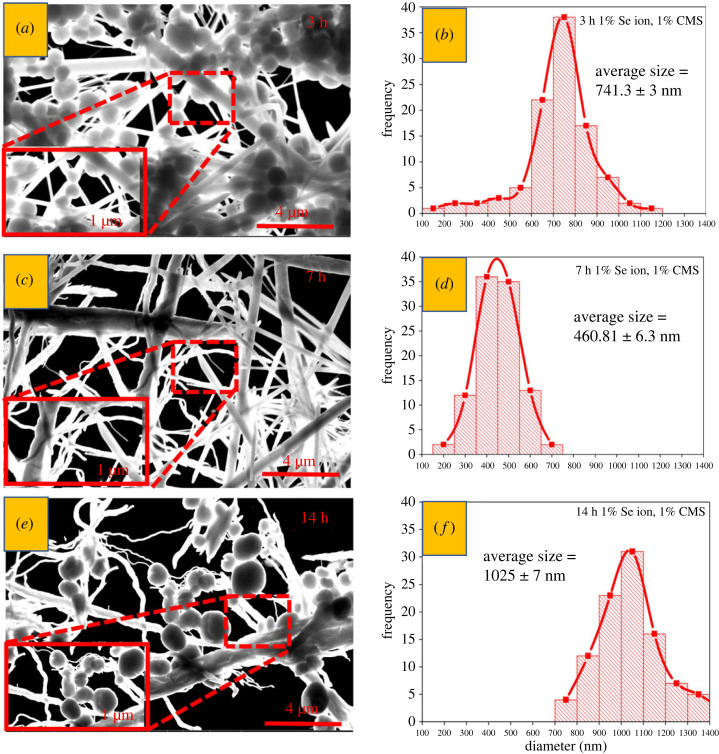
(*a,c,e*) STEM images and (*b,d,f*) size distribution of selenium nanorods prepared from 1% of Na_2_SeO_3_ and 1% of CMS at 160°C after (*a,b*) 3 h, (*c,d*) 7 h and (*e, f*) 14 h reaction time. The red dotted rectangles show the rods' details.

The size distribution plots in figure 4 were determined from STEM images presented in the same figure. The average width was obtained by counting 100 randomly selected rod widths from each condition. A Gaussian distribution fit yielded the mean size of (741.3 ± 3) nm, (460.81 ± 6.3) nm and (1025 ± 7) nm for 3, 7 and 14 h reaction times, as shown in figure 4, respectively. The data indicate that moderate-size nanorods are formed after a 7 h reaction compared with the 3 h and 14 h, correlating well with the DLS measurements (electronic supplementary material, figure S2). A possible explanation might be the partial aggregation of the selenium nanostructures at longer times (14 h) reaction. However, the average width of nanorods was found lower after 3 h and higher after 14 h compared with the 7 h reaction time (figure 4). In hydrothermal reactions, the degree of supersaturation plays a vital role in nucleation and crystal growth. Alongside intraparticle growth, Ostwald ripening is also essential. As time passes, the degree of supersaturation decreases, leading to the growth of nanostructures to a larger size. However, we hypothesized that when nanorods reach a minimum size, the residual CMS and sodium selenite may reach equilibrium due to the slow-reducing tendency of CMS. Later, the nanorods begin to agglomerate again due to the continuous Brownian motion of molecules, increasing the apparent size. The sodium peak indicates that the highest SMCA content was detected in the smallest nanoparticles (7 h, figure 3*f*), but it was then practically entirely eliminated (14 h, figure 3*i*). The size differences might also be caused by the residual CMS in the supernatant wrapped around the nanorods as a capping agent through its active functional groups, increasing their apparent thickness.

**Figure 5.**
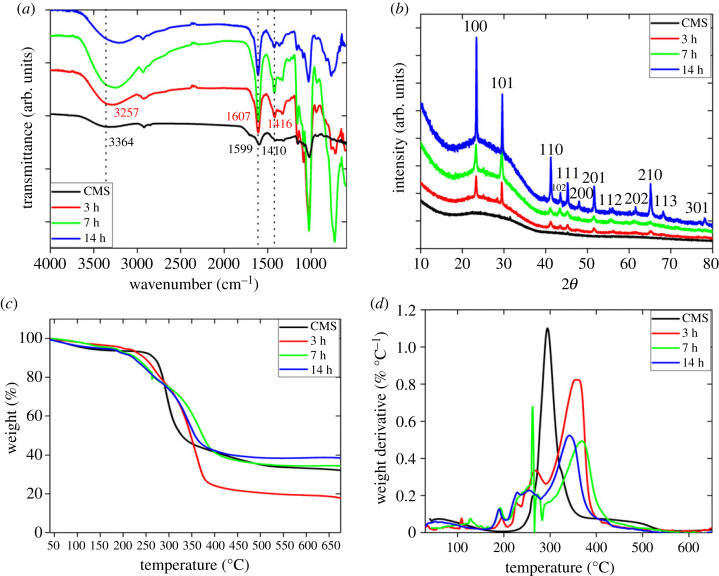
Structural analysis of CMS and selenium nanostructures (1% Na_2_SeO_3_ and 1% CMS at 160°C) after different reaction times. (*a*) ATR-FTIR, (*b*) X-ray diffraction, (*c*) TGA and (*d*) differential thermogravity (DTG) of selenium nanostructures.

FTIR spectroscopy was chosen to evaluate the molecular interactions between CMS, elemental and ionic selenium. The displacement, appearance or disappearance of bands in the FTIR spectra may be related to the interactions of CMS with selenium nanostructures. Figure 5*a* shows the FTIR spectra of CMS reacted for 3 h (black), 7 h (red) and 14 h (green). The intensity of hydroxyl (-OH, approx. 3364 cm^−1^) and carboxylic groups (-COOH, approx. 1599 cm^−1^) shifted with the reaction time. That may be evidence of selenium ion reduction to elemental selenium [37]. The blue shift of the -OH peak occurred after 3 h (red), documenting the presence of the Se-O bond. It suggests that CMS acted as the stabilizing agent during the growth [38]. The formation of selenium nanostructures was further supported by XRD (figure 5*b*). The bulk Se diffraction peaks 2*θ* are summarized in electronic supplementary material, table S1 together with their corresponding (hkl) planes and *d*-spacings [22,24,25,39,40]. The diffraction peaks at (100) and (101) planes show the trigonal crystal lattice with constants $c=4.94\overset{\circ }{A},b=4.355\overset{\circ }{A}$according to the CIF file AMCSD 0011257 [41]. The seemingly major intensity of the 100 and 101 planes in figure 5*b* indicates that selenium nanostructures tend to grow preferentially in the [001] direction. The presence of crystalline selenium proved the feasibility of CMS-directed synthesis under hydrothermal conditions (figure 5*b*).

Furthermore, the effect of reaction time on the size distribution of nanostructures evidence was supported by the classifier model, partial least squares discrimination analysis (PLS-DA), which was trained for the FTIR spectra; all FTIR spectra were used to build a PLS-DA model, which was able to classify groups based on the FTIR data. As a result, clear functional groups were detected for samples that underwent reactions lasting 3, 7 and 14 h, thanks to the similar vibrational bonding between selenium and oxygen (Se-O) created by the active sites present on the CMS (-OH, -COOH) and the selenium nanostructure produced during the same time intervals. That led to separated classes for each time point of the reactions, which indicates that time plays a crucial role in the size distribution, as shown in the electronic supplementary material, figure S7.

The thermal properties of CMS and selenium composites were investigated in a temperature ramp ranging from 50 to 700°C at a heating rate of 10.00°C min^−1^ in nitrogen, figure 5*c,d*. Heating to 700°C resulted in carbonization and ash formation. While CMS (black) loses about 40% weight at around 300°C, the reaction product after 3 h (red) loses only 25% at above 300°C (figure 5*c*). On the other hand, the samples that reacted for 7 and 14 h had almost identical residual weights as CMS (figure 5*c*). In figure 5*d*, the differential thermogravity (DTG) curves for 3 h (red), 7 h (green) and 14 h (blue) show three peaks at around 50–100°C, 200°C and 300–350°C. They correspond to water loss and degradation of selenium and CMS, respectively [33]. Apparently, the 7 h sample (green) showed higher CMS stability, with a decomposition temperature of approximately 370°C, than the other two reaction times (358 and 341°C for 3 and 14 h, respectively) or the pure CMS (294°C).

### 3.3. Effect of Se ion concentration

**Figure 6.**
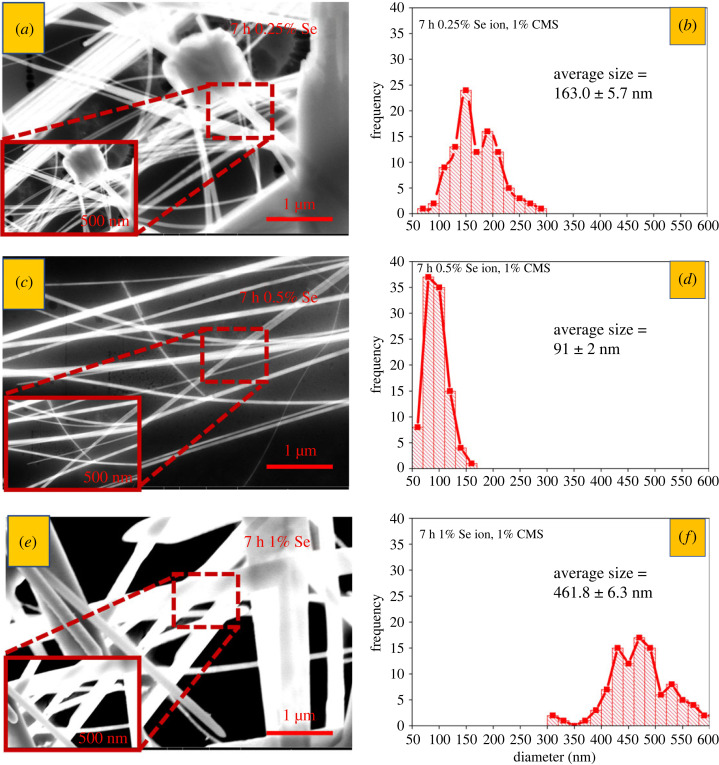
(*a,c,e*) STEM images and (*b,d,f*) size distribution of selenium nanorods with 1% CMS and different sodium selenite (Se ion) concentrations (0.25%, 0.5% and 1%) reacted for 7 h at 160°C. The red dotted rectangles show the rods’ details.

**Figure 7.**
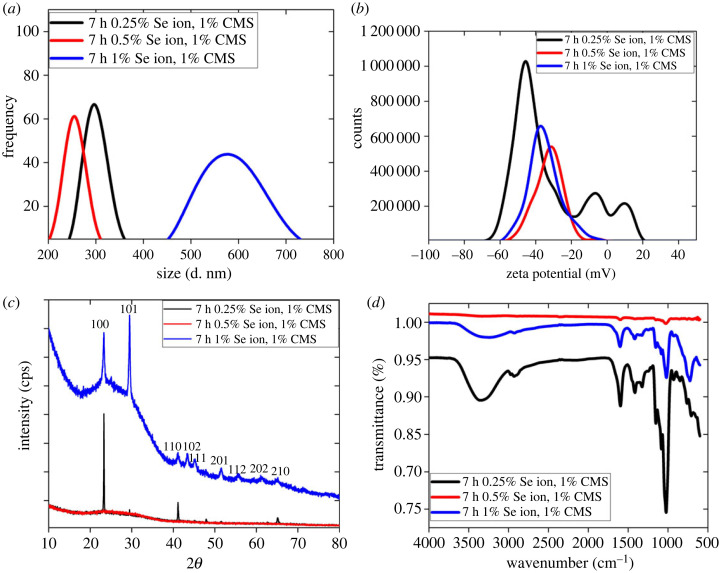
(*a*) Size distribution by DLS, (*b*) zeta potential, (*c*) XRD pattern and (*d*) FTIR spectra of selenium nanorods as a function of sodium selenite concentration after reacting for 7 h at 160°C.

The effect of Se ion concentration in the synthesis reaction also plays a crucial role in controlling the average thickness of the selenium rods, as shown in figure 6. The STEM images and size distribution analysis for the Se ion concentration dependence (0.25%, 0.5% and 1%) for a 7 h reaction at 160°C is shown in figure 6. It revealed a non-monotonic trend with the smallest nanorods obtained at medium Se ion concentration in Na_2_SeO_3_. The average nanorod diameter was established from the Gaussian fit to (163.0 ± 5.7), (91 ± 2) and (460.8 ± 6.3) nm for the 0.25%, 0.5% and 1% Se ion concentrations, respectively. That correlated with the DLS measurement (figure 7*a*). Size reduction between 0.25% and 0.5% of Na_2_SeO_3_ is probably caused by promoted nucleation due to the higher Se ion concentration. On the other hand, the large species obtained at 1% Na_2_SeO_3_ corresponded with the crystallographic change, as shown below. Another vital application aspect is dispersion stability [42]. The zeta potential measured at a constant CMS concentration and varied sodium selenite concentration scaled in the same order as the nanorod size after the reaction (figure 7*b*). The values of –32.5, –32.4 and –34.9 mV obtained for 0.25, 0.5 and 1% Na_2_SeO_3_, respectively, suggest good dispersion stability at all tested concentrations due to the electrostatic repulsion [42]. In fact, such high negative zeta potential values in the presence of interacting polymer give rise to bimodal distributions of nanostructures caused by their partial aggregation, correlating with our current observations [42]. That may explain the coexistence of nanorods and nanospheres observed after 3 h of the reaction.

**Figure 8.**
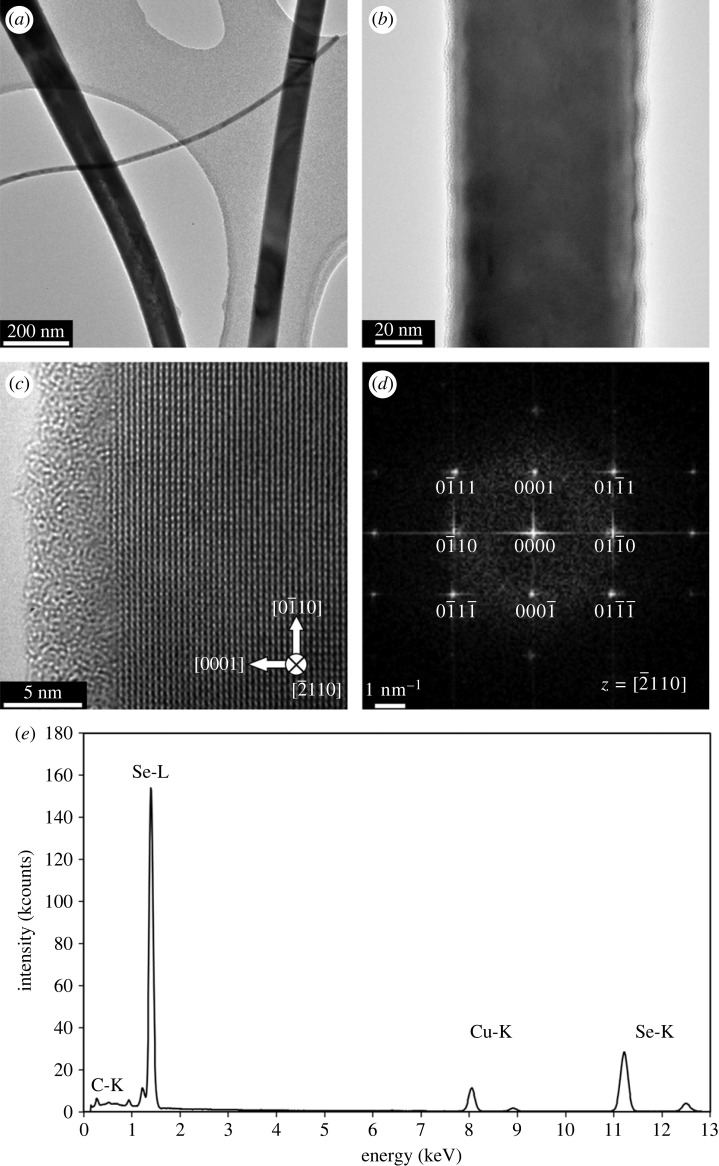
TEM analysis of Se nanorods after the reaction for 7 h at 160°C with 0.5% sodium selenite. (*a*) An overview TEM bright-field image, (*b,c*) high-resolution TEM (HRTEM) images of a single Se nanorod. The rods are covered by an amorphous film. (*d*) An analysis of a fast Fourier transformation (FFT) pattern calculated from the HRTEM image in (*c*) determined Se nanorod with P3_2_21 trigonal crystal lattice oriented to the zonal axis $\left[\bar{2}110\right]$ and growth direction in a [0001] direction. (*e*) EDS spectra were acquired from a single Se nanorod.

The XRD pattern in figure 7*c* shows the characteristic diffraction peaks of the selenium nanorods. The planes corresponded to trigonal selenium crystals [39,43]. A TEM analysis of Se nanostructures was conducted for the sample reacted for 7 h at 160°C with 0.5% sodium selenite. The TEM results presented in figure 8 reveal the sample in the form of nanorods, while the nanospheres were not determined, correlating with the SEM observations in figure 6*c*. The TEM performed in bright-field mode found that the nanorods were straight and with minimum crystal defects (figure 8*a*). Some nanorods were found with twin defects resulting in their ‘zig-zag’ growth (not presented here), but, generally, the nanorods had a single crystal structure within their length, as demonstrated in high-resolution TEM (HRTEM) images with atomic resolution in figures 8*b,c*. The crystallographic analysis in figure 8*d* was performed using fast Fourier transformation (FFT) of the HRTEM image in figure 8*c*. It determined Se nanorods with P3_2_21 trigonal crystal lattice, where the [0001] directions were parallel with a longitudinal axis of the rod, suggesting its growth direction. The crystallographic FFT data agreed with the XRD measurement and a standard listed in [41]. The EDS analysis was performed at a single nanorod, and the collected spectra in figure 8*e* indicate its pure Se composition. The minor amount of carbon may correspond with surface contamination of the nanorod (the amorphous structure at the nanorod edges in figure 8*c*), and the Cu signal comes from the copper TEM grid.

In figure 7*d*, FTIR spectra show the characteristic hydroxyl (-OH) and carboxylic (-COOH) peaks of CMS at approximately 3364 cm^−1^ and approximately 1599 cm^−1^ on the surface of all selenium nanorods, respectively (figure 5*a*). In addition, large shifts of the broadband peak for 1% of Na_2_SeO_3_ (blue) and the intensity change of the -COOH peak may indicate that more Se-O bonds are formed from the CMS hydroxyl groups (figure 7*d*). These could stabilize the nanorods and potentially explain the zeta potential variation (figure 7*b*). The XPS spectra from the C1s and O1s peaks verified the Se-O bond formation. Moreover, the -C-O-Se signal confirms that CMS behaves as a capping agent.

**Figure 9.**
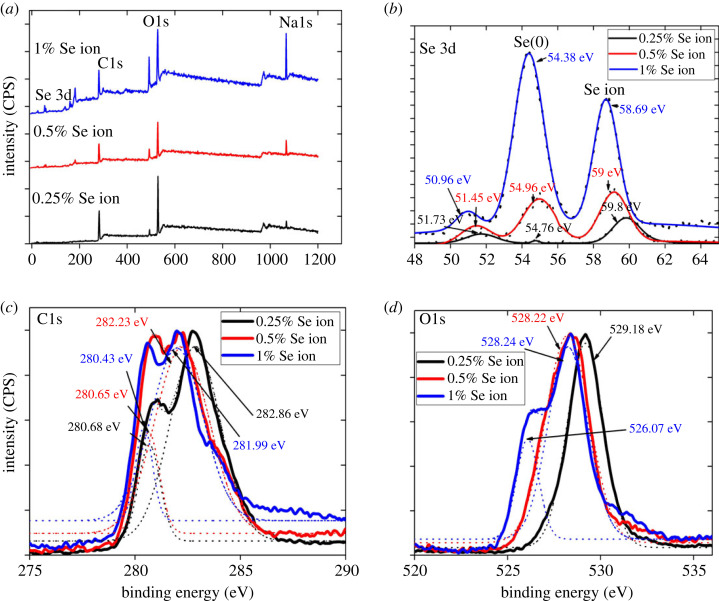
XPS spectra of selenium nanorods synthesized using different sodium selenite concentrations reacted for 7 h at 160°C: (*a*) wide spectrum, (*b*) Se3d bond, (*c*) C1s and (*d*) O1s detailed spectra.

The Se (3d) XPS spectrum in figure 9*b* compares the selenium valence state in nanorods prepared at different sodium selenite concentrations. The Se(3d) peak consists of two subpeaks, Se 3d5/2 and Se 3d3/2. Values from the literature suggested that they are separated by 0.86 eV [44–46]. During Gaussian fitting, we defined the parameters and found that it is necessary to fit the subpeaks. According to the literature, Se(0) is expected to appear at the binding energy of approximately 55.4 eV, Se(IV) at 59.5 eV, Se(VI) at 61 eV and Se(-II) at less than 55 eV [47,48]. Therefore, all the samples in figure 9*b* showed Se(0) along with Se(IV), representing either residual unreacted reagents or polarized selenium from the outer layer charged again due to the interaction with the capping agent. One per cent Se ion (blue) showed the highest intensity of Se(0) relative to Se(IV), marking the highest efficiency of the selenium reduction.

All three samples displayed two C1s peaks (figure 9*c*) attributed to C–C (280.43–280.68 eV) and C–C=O (281.9–282.86 eV) [36,49]. That is yet another evidence of the CMS presence on the nanorod surface. Moreover, the 1% Na_2_SeO_3_ sample had lower binding energy than the 0.5% and 0.25% samples. A possible reason could be more capping sites of CMS available for forming the Se-O bond, which is also supported by the higher negative zeta potential (figure 7*b*). As the concentration of selenium ions increases from 0.25% to 1%, the binding energy of oxygen decreases from 529.18 to 528.24 eV (figure 9*d*). Moreover, an additional peak is formed as a shoulder at 526.0 eV for 1% Na_2_SeO_3_, possibly due to the carbonyl oxygen's interaction with selenium nanorods. The binding energy of Na (1s) in electronic supplementary material, figure S9 also depicts the presence of sodium in carboxymethyl starch and the presence of residual sodium selenite, traces of which are also present in the Se (3d) spectrum in figure 9*b*.

Complementary experiments revealing analogical nanorod size dependence on the Na_2_SeO_3_ concentration are available in the supporting information. It includes STEM images and size distributions (electronic supplementary material, figure S3), FTIR, XRD and hydrodynamic size obtained by DLS (electronic supplementary material, figures S4 and S5). Given all the experimental results, we suggest that the nanorod shape is directly related to using CMS as the capping and reducing agent and that different polymers may yield nanostructures with different morphology.

### 3.4. Reaction temperature

**Figure 10.**
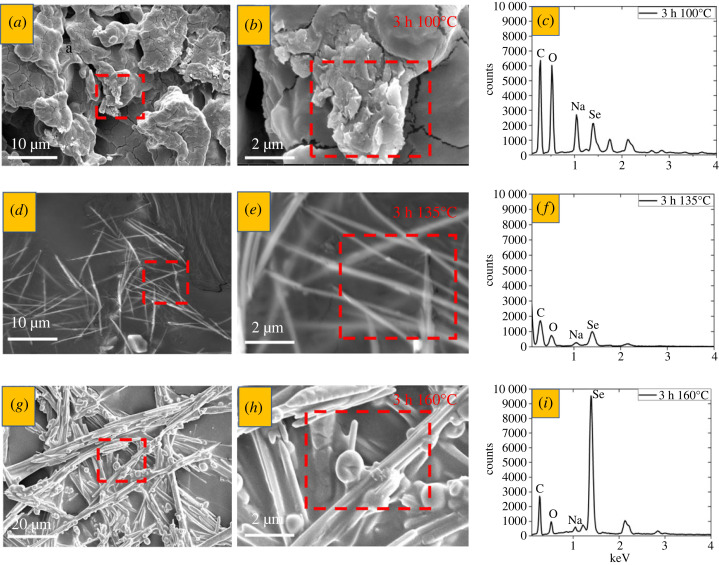
SEM-EDS analysis of selenium nanostructures (spheres, rods) with 1% of sodium selenite and 1% of CMS: (*a,d,g*) lower and (*b,e,h*) higher magnification SEM images, and (*c,f, i*) EDS spectra for samples reacted for 3 h at (*a,b,c*) 100, (*d,e,f*), 135 and (*g,h,i*) 160°C. Red rectangles show the area of larger magnification (*a,d,g*) or EDS spectra collection (*b,e,h*).

The temperature effect on the growth of selenium nanostructures was observed for 1% of CMS and 1% of Na_2_SeO_3_ reacting for 3 h at 100, 135 or 160°C. SEM-EDS analysis of the formed nanostructures is captured in figure 10. No clear evidence exists for selenium nanostructures growing on CMS at 100°C, figure 10*a,b*. Instead, micron-sized objects were formed with a high carbon and oxygen content, as documented by EDS spectra in figure 10*c*. On the other hand, nanostructures were formed at 135°C (figure 10*d,e*) and 160°C (figure 10*g,h*). Apparently, the threshold temperature lies between 100 and 135°C. Supposedly, more free carbonyl and hydroxyl groups are available to reduce the selenium ions into the Se(0) at higher temperatures. These results correlate with the DLS data (electronic supplementary material, figure S6*a*). A statistical relation between the size distribution obtained from STEM and DLS is shown in electronic supplementary material, figure S10. Moreover, a surface plasmon resonance (SPR) peak was detected in the UV-VIS spectra between 390 only for (blue) 3 h 160°C shown in electronic supplementary material, figure S6*b* [50]. The SPR position relates to nanoparticle size such that smaller nanoparticles absorb at longer wavelengths. Therefore, a redshift to higher wavelengths indicates smaller particles [51]. Aggregated particles provide no surface plasmon resonance due to the defects and irregularities in their structure.

FTIR spectra confirmed the formation of selenium nanostructures at 135 and 160°C by the increased hydroxyl (-OH, approx. 3364 cm^−1^) and carboxylic (-COOH, approx. 1599 cm^−1^) absorption intensities (electronic supplementary material, figure S6*c*). Moreover, electronic supplementary material, figure S6*d* shows the XRD patterns of selenium nanostructures synthesized at different temperatures. The 2*θ* peaks of selenium nanorods reacted at 160°C found at 23.55°, 29.72°, 41.27°, 43.68°, 45.43°, 47.1° 51.72°, 56.07° and 65.24° were indexed as the 100, 101, 110, 102, 111, 200, 201, 112 and 210 planes. On the other hand, no crystallites were detected for 100 and 135°C reactions.

**Figure 11.**
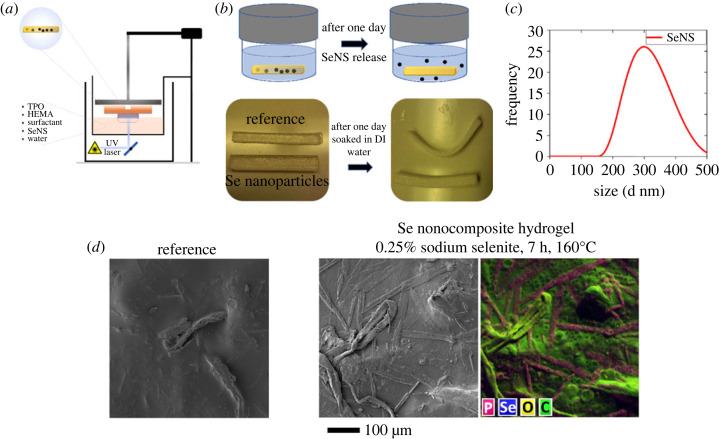
(*a*) Vat photopolymerization three-dimensional printing scheme of the Se-loaded hydrogels, (*b*) design of the particle release experiment and photos of the samples with a pronounced bending of the reference (without Se) detected after the submersion, (*c*) DLS confirmation of the released nanostructures' size, (*d*) SEM images of the reference (without Se nanostructures) and sample doped with Se nanostructures (0.25% sodium selenite, 7 h, 160°C) including an EDS map.

Finally, a pilot test was conducted to verify the potential application of the selenium nanostructures and their release from a hydrogel carrier (figure 11). A purified supernatant obtained by reacting 0.25% sodium selenite and 1% CMS for 7 h at 160°C was added to a HEMA-based photopolymer resin and three-dimensional printed with a vat photopolymerization three-dimensional printer (figure 11*a*). HEMA is a water-soluble monomer commonly used in biomedical applications, such as contact lenses. The HEMA-based resin could be mixed with water or aqueous solutions to cure into a hydrogel. The printed body was submersed into the water for 21 h (figure 11*b*), and the released nanostructures were confirmed by DLS (figure 11*c*). However, the selenium nanoparticles interacted with the TPO photoinitiator and caused it to form micron-sized anisotropic structures, as documented by the presence of phosphorus in the EDS map (figure 11*d*). That probably stiffened the hydrogel and reduced the level of bending observed after the submersion (figure 11*b*). A detailed mechanism of the interaction and its potential impact on biocompatibility is not yet known. Nevertheless, we consider this a proof of concept and a good starting point for investigating bio-three-dimensional printing and other biomedical applications with the selenium nanoparticles synthesized by the presented method.

## 4. Conclusion

A detailed study on the selenium nanorods and nanospheres synthesis by the hydrothermal technique using carboxymethyl starch (CMS, DS = 0.3) as a reducing and capping agent is reported. An in-depth characterization (SEM, STEM, XRD, FTIR, DLS, XPS) of the particle size and structure regarding the sodium selenite concentration, reaction time and temperature is also provided. The minimum temperature required for selenium reduction and nanostructure formation was between 100 and 135°C. A substantial increase in size was detected at prolonged reaction times. EDS and XPS results confirmed that the selenium nanostructures contain Se(0) and Se–O bond states, with CMS as a capping agent on the particle surface. That was further supported by FTIR and zeta potential measurements. The smallest nanorods (average size of 91 nm) were obtained for 0.5% sodium selenite, and 1% CMS reacted for 7 h at 160°C, while the XRD patterns revealed that the nanorods have a trigonal phase. A selenium nanocomposite hydrogel was prepared by a vat photopolymerization three-dimensional printing and tested for nanoparticle release when introduced to water, as confirmed by DLS measurement. That verified the potential of the presented nanoparticles to be included in complex structures and delivered on an application site by hydrogel carriers. Such behaviour promises excellent potential for bio-three-dimensional printing and other biomedical applications.

## Data Availability

Our raw data are deposited in Dryad and are available to the public via the following link, [52] https://doi.org/10.5061/dryad.bnzs7h4gs. Supplementary information is available with the manuscript. The data are provided in electronic supplementary material [53].
